# Neuropsychiatric and neurological problems among Vitamin B12 deficient young vegetarians

**DOI:** 10.17712/nsj.2017.3.20160445

**Published:** 2017-07

**Authors:** Aneel Kapoor, Mukhtiar Baig, Saeed A. Tunio, Abdul S. Memon, Hotchand Karmani

**Affiliations:** *From the Department of Biochemistry (Kapoor, Memon), Muhammad Medical College, Mirpurkhas, from the Department of Biochemistry (Tunio), Khairpur Medical College, Khairpur, Sindh, from the Department of Clinical Biochemistry (Baig), Faculty of Medicine, Rabigh, King Abdulaziz University, Jeddah, Kingdom of Saudi Arabia, and from the Department of Neurology (Karmani), Khulla Hospital, Muscat, Oman*

## Abstract

**Objective:**

To assess the frequency of neuropsychiatric and neurological problems in apparently healthy young vegetarians and estimate serum Vitamin B12, methylmalonic acid (MMA), and folic acid levels.

**Methods:**

This prospective study was conducted in the Department of Biochemistry, Basic Medical Sciences Institute (BMSI), Jinnah Postgraduate Medical Center (JPMC), Karachi, Pakistan, in the years of 2012 and 2013. The data of 100 vegetarians and 100 omnivores were analyzed and compared.

**Results:**

The serum concentration of Vitamin B12 was significantly lower in the vegetarian group compared with the omnivore group (238±71 pg/ml vs. 401±170 pg/ml, p<0.001). In the vegetarian group, MMA level was significantly higher compared with the omnivores (285±89.4 nmol/L vs. 191±40.5 nmol/L, p<0.001). Regarding the neuropsychiatric and neurologic problems in the vegetarian group, the frequency of depression was 31% compared with 12% in the omnivore (p=0.002), paresthesias were 11% compared with 3% in the omnivores (p=0.04), peripheral neuropathy was 9% compared with 2% in the omnivores (p=0.05), psychosis was found in 11% subjects compared with 3% in the omnivores (p=0.04).

**Conclusion:**

Vegetarians have Vitamin B12 deficiency and are more prone to developing neuropsychiatric and neurological problems.

Nutrition is the main determinant of human mental performance, especially during aging and Vitamin B12 is linked to the psychiatric disorders, including impaired memory, irritability, dementia, and depression.^1^ Vitamin B12 has a vital role in DNA synthesis during cell division.^2^ It is also associated with the production of dopamine and serotonin (neurotransmitters).^3^ Dietary sources of Vitamin B12 are mainly of animal origin, including meats, dairy products, and eggs.^4^ Hence, Vitamin B12 deficiency is more common in vegetarians and vegans.^5^ Vitamin B12 plays several very important functions in the human body. It is required as a coenzyme for the myelin synthesis in the nervous system, and it facilitates the Methionine-Homocysteine Cycle.^4^

Recently, Ssonko et al^6^ (2014) described that the lack of Vitamin B12 is frequent among hospitalized psychiatric patients and no hematological findings are found in the majority of patients. Some studies have put at between 5% and 30% the prevalence of Vitamin B12 deficiency among psychiatric inpatients.^7^,^8^ Furthermore, psychiatric illnesses may cause Vitamin B12 deficiency by affecting patients’ appetite, diet, or metabolic requirement.^9^ Recently, a study in Pakistan showed 57% prevalence of Vitamin B12 deficiency, after excluding strict vegetarians from their study.^10^ This high prevalence of Vitamin B12 deficiency in omnivores is not only surprising but also alarming. Most of the studies related to Vitamin B12 deficiency have been conducted among the elderly but, in our study, the subjects were comparatively young (age ranges from 20 to 40 years old).

Numerous studies have reported the prevalence of Vitamin B12 deficiency among vegetarians and psychiatric inpatients. Our study is novel in the sense that we measured this vitamin in apparently healthy subjects and found neuropsychiatric and neurological problems among Vitamin B12 deficient young vegetarians. Moreover, there are no previous studies evaluating these 2 vitamins and neuropsychiatric problems in Tharparker, Sindh, Pakistan. The present study aimed to assess the frequency of neuropsychiatric and neurological problems in apparently healthy vegetarians and to estimate Vitamin B12, MMA, and folic acid levels in the local population. The present study has been carried out in Mithi, District Tharparker (Sindh), because a large number of the population in Mithi District belong to the Hindu community and most of the people of this community adhere to a vegetarian diet because of family conventions or religious reasons.

## Methods

The present cross-sectional study was conducted in the Department of Biochemistry, BMSI, JPMC, Karachi, Pakistan, in the years of 2012 and 2013. Four hundred subjects were assessed for this study and, finally, the data of 200 apparently healthy volunteers (age ranging from 20-40 years) was analyzed, and all subjects were recruited for this study from the general population of Mithi, District Tharparkar, Sindh. We divided them into 2 groups according to their dietary pattern (100 subjects were vegetarians, and 100 were omnivores). Out of 200 subjects, 149 (74.5%) were males and 51 (25.5%) were females. The vegetarian group had adhered to a vegetarian diet since their childhood. Except for the small consumption of dairy products, they ate no animal sourced food such as meat, poultry, fish, or eggs.

The omnivore group ate all types of food, including vegetables and animal foods such as meat, poultry, fish, and eggs. All study subjects were apparently healthy and had no physician-diagnosed disease (especially inflammatory disease). A written consent was taken from all participants. Each participant, on the day of recruitment, filled in a detailed questionnaire about the demographic and medical information. The questionnaire also included information about the participant’s physical activity, blood pressure, dietary pattern, smoking habits, and milk consumption. We excluded the subjects from this study if they were taking multivitamins, anti-convulsants, pregnant and lactating females, alcohol users, diabetics, cigarette smokers, having gastrointestinal and others causes of the spinal cord and peripheral nerve diseases.

All subjects were examined for neuropsychiatric problems by the neurologist, psychiatrist, and general physician. All subjects were screened for dementia by Mini-Mental Status Examination (MMSE) (dementia, MMSE score <24). The diagnosis of dementia was established by Diagnostic and Statistical Manual criteria. The Hamilton Rating Scale for Depression was used for evaluating the severity of depression.^11^ Moreover, all those patients suspected to have cognitive impairment underwent a detailed assessment of various cognitive domains by a neuro physician. The ethical approval was taken from the Research Ethics Committee of the BMSI, JPMC, Karachi (F.1-2BMSI-E.COMT/JPMC) and the study was performed according to the principles of the Helsinki Declaration.

The blood samples were collected (5 milliliters) from each participant after an overnight fasting. The serum was separated and stored at -70 0C for Vitamin B12, folic acid, and MMA analysis. Serum Vitamin B12 and folic acid were estimated by Immulite 1000, using kits manufactured by (Siemens Healthcare Diagnostics Products, Ltd., Sudbury, UK). Serum MMA was estimated by ELISA kit manufactured by Equipar, Belgium. The coefficient variation (CV) for Vitamin B12 was 2.9%, folic acid was 5.1%, and MMA was 7.4%. The biochemical Vitamin B12 deficiency was considered at a level below <200 pg/ml, and folic acid deficiency at <3 nmol/L while MMA level >271 nmol/L was considered higher. The criteria for diagnosing Vitamin B12 deficiency was “if serum cobalamin level <150 pmol/L and MMA >0.4 µmol/L (in the absence of renal failure and folate deficiency)”.^12^

### Statistics

Data was analyzed with Statistical Package for the Social Sciences version 16 (SPSS Inc, Chicago, IL, USA). Values of the qualitative variables are represented by frequency and percentages while quantitative variables are presented by mean with standard deviation and the comparison between groups and subgroups was carried out by applying student t-test and Fisher’s exact test. Significance was taken at *p*-value <0.05.

## Results

The comparison of mean values of baseline characteristics is given in **Table 1**. The serum concentration of Vitamin B12 was significantly lower in the vegetarian group compared with the omnivore group (238±71pg/ml vs. 401±170 pg/ml, *p*<0.001) and the folic acid level was significantly lower in the vegetarian group compared with the omnivore group (16.1±4 nmol/l vs. 25.1±7 nmol/l, *p*<0.001). Nevertheless, there was no folic acid deficiency in both groups. In the vegetarian group, MMA level was significantly higher compared with the omnivores (285±89.4 nmol/L vs. 191±40.5 nmol/L, *p*<0.001). There were 77% male and 23% female in the vegetarian group while 72% male and 28% female were in the omnivore group (**Table 1**).

**Table 1:** Comparison of variables in vegetarians and omnivores groups.

Variables	Vegetarians (n=100)	Omnivores (n=100)
Age (years)	27.7±5.85	28.8±5.70
***Gender***
Male	77 (77)	72 (72)
Female	23 (23)	28 (72)
Vitamin B12(pg/ml)	238±71	401±170*
Folate (nmol/L)	16.1±4	25.1±7*
MMA (nmol/L)	285±89.4	191±40.5*

Values are expressed as mean±standard deviation, N - number of subjects,

* *p*<0.001 compared vegetarians with omnivores, MMA - Methylmalonic acid

In the vegetarian group 51 (51%) subjects had Vitamin B12 level less than cut-off value (200 pg/ml) and in the omnivores group, only 03 (3%) subjects had Vitamin B12 deficiency (*p*<0.001) while folic acid was not deficient in both groups. In the vegetarian group, 44% of the subjects had a high level of MMA (>271 nmol/L) while 56% had its normal levels (100-271 nmol/L). However, in all the omnivore subjects, MMA level was within the normal range. Gender-wise distribution showed that, among the vegetarian group, 61% of females were suffering from Vitamin B12 deficiency compared with 48% of males (*p*<0.001). Furthermore, when Vitamin B12 sufficient vegetarians compared with Vitamin B12 sufficient omnivores, a significantly higher number of omnivores had B12 >200 pg/ml (*p*<0.001) (**Table 2**).

**Table 2:** Gender-wise comparison of normal and low levels of serum vitamin B12 and folic acid and normal and higher levels of MMA.

Variables	Male n=149 G-I n=77, G-II n=72	Female n=51 G-I n=23, G-II n=28	Total n=200
***B12 (<200pg/ml)***			
G-I	37 (48.1)*^†^	14 (60.9)^α^	51 (51)^∞^
G-II	03 (4.2)	0 (0)^‡^	03 (3)
***B12 (>200pg/ml)***			
G-I	40 (51.9)*^†^	9 (39.1)^α^	49 (49)^∞^
G-II	69 (95.8)	28 (100)^‡^	97 (97)
***Folate <3nmol/L)***			
G-I	0 (0)	0 (0)	0 (0)
G-II	0 (0)	0 (0)	0 (0)
***Folate (>3nmol/L)***			
G-II	100 (100)	100 (100)	100 (100)
G-II	100 (100)	100 (100)	100 (100)
***MMA (100-271nmol/L)***			
G-I	44 (57.1)*^†^	12 (52.2)^α^	56 (56)^∞^
G-II	72 (100)	28(100)^‡^	100 (100)
***MMA (>271nmol/L)***			
G-I	33 (42.9)	11(47.9)^α^	44 (44)^∞^
G-II	0 (0)	0 (0)	0 (0)

MMA - Methylmalonic acid, G-I - group I (Vegetarian), G-II - group II (Omnivores), N - Number of subjects, % - Percentage,

* *p*<0.001 when male vegetarians’ compared with male omnivores,

α *p*<0.001 when female vegetarians’ compared with female omnivores,

† *p*<0.001 when male vegetarians’ compared with female vegetarians’,

‡ *p*=0.13 non-significant when male omnivores compared with female omnivores,

∞ *p*<0.001 when total vegetarians’ compared with total omnivores

**Table 3** shows the comparison of the few neuropsychiatric and neurologic problems in the vegetarian and omnivore groups. In the vegetarian group, the frequency of depression was 31% compared with 12% in the omnivores (*p*=0.002), paresthesias was 11% compared with 3% in omnivores (*p*=0.04), and peripheral neuropathy was 9% compared with 2% in the omnivores (*p*=0.05). Among the vegetarians, psychosis was found in 11% of the subjects compared with 3% among the omnivores (*p*=0.04), memory impairment was 7% compared with 2% in the omnivores (*p*=0.17), and the personality changes were 5% compared with 1% in the omnivores (*p*<0.01).

**Table 3:** Comparison of neurologic and psychiatric problems between vegetarians and omnivores.

Neurologic and psychiatric problems	Vegetarians	Omnivores	*P*-value
	n (%)		
Paresthesias	11 (11)	3 (3)	0.04
Peripheral neuropathy	09 (09)	2 (2)	0.05
Personality change	05 (05)	1 (1)	0.21
Mild memory impairment	07 (07)	2 (2)	0.17
Depression	31 (31)	12 (12)	< 0.002
Psychosis	11 (11)	03 (3)	0.04
**Total**	**74 (74)**	**23 (23)**	**<0.001**

N - number of subjects

## Discussion

In our study, the vegetarian subjects had significantly lower mean values of Vitamin B12 compared with the omnivores. These results are compatible with several other studies.^13^,^14^ In the present study, Vitamin B12 deficiency was more common in female participants as compared with males. This result is inconsistent with several other studies,^6^,^15^ which observed Vitamin B12 deficiency more in males. There could be several reasons for this difference like sample size, type of population, and the age of the participants.

The current study found that the neuropsychiatric and neurological problems like depression, paresthesias, peripheral neuropathy, psychosis, memory impairment and personality change were more common in vegetarians. These results are incongruent with other studies.^16^-^17^ These changes could be because of the deficiency of Vitamin B12 in the vegetarians. It seems that there is a relationship between the deficiency of Vitamin B12 and neuropsychiatric and neurological problems but not with folic acid. Jayaram et al^18^ reported the psychiatric symptomatology in Vitamin B12 deficient subjects and described that 58% had paranoid schizophrenia, 16% undifferentiated schizophrenia, 5% had episodic psychosis, 5% bipolar affective disorder, and 15.8% depressive disorders. In that study, 74% of the total subjects were vegetarians, and 68% were females. Recently, Dahal et al^19^ reported a significantly lower level of Vitamin B12 in dementia.

Neurological manifestations due to Vitamin B12 deficiency are attributable to the pathological changes in the peripheral nerves, optic nerves, and posterior and lateral columns of the spinal cord and the brain. These changes result in neuropsychiatric abnormalities such as neuropathy, myelopathy, myeloneuropathy, dementia and cognitive impairment, cerebellar ataxia, optic atrophy, and psychosis and mood disturbances.^20^

In contrast to our results, Michelakos et al^21^ reported that cognitive impairment was associated with low folate levels in males. Their meta-analysis showed an adverse effect of the low folate levels on cognition while the Vitamin B12 was non-significantly associated.^21^ It has been reported that lower levels of vitamin B12 and folate and higher levels of plasma HCY are related to cognitive impairment in elderly individuals^22^ but, in our study, we found no significant difference in plasma folic acid levels. It might be because our study group was vegetarian and comparatively young. Our results demonstrate that Vitamin B12 insufficiency is common for vegetarians and, additionally, neuropsychiatric and neurological problems are common in this group. Initially, Vitamin B12 deficiency and its related problems are silent until a serious consequence occurs. Therefore, it is recommended that people should eat a diet fortified with Vitamin B12 and folic acid to prevent these nutrient deficiencies, particularly in vegetarians. Early detection of Vitamin B12 deficiency is necessary for preventing potentially irreversible neuropsychiatric changes. So it is recommended that, in vegetarians with neuropsychiatric problems, their Vitamin B12 levels should be checked and treated if it is deficient to prevent irreversible neurological loss.

There are several limitations to the present study. Firstly, it is a cross-sectional study with a small sample size. Secondly, Vitamin B12 metabolite and other related parameters were not estimated and, thirdly, complete blood picture is not mentioned.

In conclusion, it appears that the majority of the vegetarians had vitamin B12 deficiency and they also had neuropsychiatric problems. It could be assumed that Vitamin B12 deficiency is one of the contributing factors in neuropsychiatric problems in vegetarians. Therefore, early replacement of Vitamin B12 is suggested for preventing such catastrophic damages in this high-risk group.
